# Theta Burst Stimulation Enhances Connectivity of the Dorsal Attention Network in Young Healthy Subjects: An Exploratory Study

**DOI:** 10.1155/2018/3106918

**Published:** 2018-03-13

**Authors:** Lubomira Anderkova, Dominik Pizem, Patricia Klobusiakova, Martin Gajdos, Eva Koritakova, Irena Rektorova

**Affiliations:** ^1^Applied Neuroscience Research Group, Central European Institute of Technology – Masaryk University (CEITEC MU), Brno, Czech Republic; ^2^Multimodal and Functional Neuroimaging Research Group, Central European Institute of Technology – Masaryk University (CEITEC MU), Brno, Czech Republic; ^3^Institute of Biostatistics and Analyses, Faculty of Medicine, Masaryk University, Brno, Czech Republic

## Abstract

We examined effects of theta burst stimulation (TBS) applied over two distinct cortical areas (the right inferior frontal gyrus and the left superior parietal lobule) on the Stroop task performance in 20 young healthy subjects. Neural underpinnings of the behavioral effect were tested using fMRI. A single session of intermittent TBS of the left superior parietal lobule induced certain cognitive speed enhancement and significantly increased resting-state connectivity of the dorsal attention network. This is an exploratory study that prompts further research with multiple-session TBS in subjects with cognitive impairment.

## 1. Introduction

Transcranial magnetic stimulation (TMS) is a noninvasive method which generates a brief strong magnetic field around a coil that induces electric currents in underlying neuronal tissue. Using this technique in repetitive way—repetitive TMS (rTMS)—we can study and influence brain plasticity in humans in vivo. Noninvasive brain stimulation (NIBS) techniques such as rTMS can be further combined with a variety of neuroimaging and electrophysiological methods which can inform subsequent NIBS, providing information about where, when, and how to stimulate the brain. Moreover, neuroimaging and electrophysiology can provide indices of neuronal activity, which make it possible to assess the changes caused by NIBS and the neural underpinnings of its behavioral aftereffects [1, 2].

The main goal of the current study was to assess immediate effects of a short rTMS session on both cognitive task performance (namely the Stroop task behavioral measures) and the changes in resting-state functional connectivity particularly within the dorsal attentional network (DAN), that is, the major large-scale brain network related to goal-directed behaviors such as visual attention tasks [3]. Stroop task is a cognitive visual task, aimed at measuring cognitive speed and executive function (inhibitory cognitive control over conflicting situation) that in fMRI studies shows involvement of anterior (mainly frontal) and posterior (mainly parietal) brain regions [4, 5]. We specifically focused on the Stroop task performance since it has been altered in early Alzheimer's disease (AD) patients [6, 7] as well as Parkinson's disease (PD) patients [8] and found to be associated with a degree of inhibition of cortical acetylcholinesterase activity by donepezil [9]. Using repeated rTMS in these patient groups may be of clinical relevance [10, 11], and exploring its potential therapeutic effect has been our major research focus [7, 12–15].

More specifically, we examined effects of rTMS using two different theta burst stimulation (TBS) protocols [16] applied over two stimulation sites in 20 healthy young subjects (HYS). We selected TBS protocols because it has been shown that TBS induces effects on excitability when applied over the motor cortex [16]. Moreover, TBS can modulate cognitive functions [17] and other behavioral functions [18], and it might have an effect on functional connectivity of major cognitive control networks [19]. Compared to classical rTMS protocols, TBS is relatively short with good participants' compliance.

Previously, rTMS studies in healthy controls showed that excitatory repetitive TMS (rTMS) increased dopamine release in the striatum [20, 21], while inhibitory protocols such as continuous TBS (cTBS) decreased striatal dopamine release and impaired performance in the cognitive task [17]. We selected two stimulation sites based on the fMRI Stroop task meta-analysis [5] including the right inferior frontal gyrus (rIFG) and the left superior parietal lobule (lSPL). Moreover, both regions are engaged in the dorsal attention network (DAN) which was our network of interest (see above). Our choice of the rIFG was based on its key role in inhibition [22] and cognitive processes that are important for the Stroop test performance. Moreover, this area seems to be involved in the hyperdirect pathway connecting the subthalamic nucleus (STN) with cortical regions engaged in executive functioning and attention processes [23]. Our group previously studied this area using classical TMS protocols and behavioral Stroop task in patients with neurodegenerative brain diseases [7, 14]. We did not want to interfere with the language area located in the left IFG (this is why we focused on the right hemisphere). The DLPFC has already been a quite heavily studied area while only few studies have targeted the IFG so far. The superior parietal lobule is involved in aspects of attention (spatial attention) and visuospatial perception, including the representation and manipulation of objects, and it is also involved in the Stroop task [5]. In our pilot fMRI data analysis of the currently used Stroop task (unpublished data), we observed more task-induced activation on the left side.

We hypothesized that excitatory (intermittent) TBS [16] will induce measurable behavioral changes that will be accompanied by distinct resting-state fMRI (rs-fMRI) changes. We did not have any strong hypothesis regarding cTBS of the IFG or SPL; however, this protocol exerts inhibitory effects (i.e., opposite effects compared to iTBS) when applied over the primary motor cortex. The work was designed as an exploratory study in order to prompt further research using a multiple-session design in subjects with mild cognitive impairment due to neurodegenerative brain diseases such as AD or PD.

## 2. Materials and Methods

Twenty young right-handed HYS participated in the study (mean age 25.2 ± 2.7 years, men/women ratio: 7/13). Exclusion criteria were any diagnosed psychiatric or neurological disorder or a cognitive deficit based on the results of a detailed neurocognitive battery performed prior to the study entry [7]. T1 MPRAGE (TR 2300 ms; TE 233 ms; voxel size 1 × 1 × 1 mm; FoV read 224 mm, FoV phase 252 mm; base resolution 256; 240 slices; gap 0.5 mm) and T2 FLAIR MRI sequences (TR 6000 ms; TE 387 ms; voxel size 1 × 1 × 1 mm; FoV 256 mm; base resolution 256; 192 slices) [24] were performed using a 3T Siemens Prisma machine and visually inspected by a clinician to exclude any structural brain pathology. Each participant signed an informed consent form, and the study was approved by the local ethics committee.

Based on literature [5] and as mentioned above, we targeted the right inferior frontal gyrus (rIFG; 46 14 32) and the left superior parietal lobule (lSPL; −24 −68 48) using a frameless stereotaxy neuronavigation with Brainsight 2, and very short excitatory (intermittent) TBS (iTBS; 190 s duration, 600 pulses) and inhibitory (continuous) TBS (cTBS; 40 s duration, 600 pulses) protocols [16] using Deymed DuoMAG XT stimulator with 70BF-Cool coil at 80% of individual AMT intensity. Magnetic stimulation was given in the room right next to the scanner over the abovementioned areas using a hand-held figure of eight coils (70 mm standard coil) placed tangentially to the scalp with the handle pointing ventrally. We used a crossover design, and the order of stimulation protocols and sites was randomised across subjects and sessions. The individual stimulation sessions were separated by at least a one-day interval without stimulation. Each session consisted of the prestimulatory fMRI session, a TBS session, and the poststimulatory fMRI session (identical to the prestimulation one).

For the purpose of this preliminary study, we were interested in behavioral outcomes, that is, response times (RT) and error rates for congruent and incongruent stimuli of the Stroop task [25] which was performed inside the scanner (TR 2050 ms; TE 35 ms; voxel size 3 × 3 × 3.5 mm; FoV 192 mm; base resolution 64; flip angle 70°; 35 slices; 165 scans; iPAT 2) and in the neural correlates of the TBS-induced behavioral changes as measured by rs-fMRI. We acquired 200 rs-fMRI scans using a gradient-echo echo-planar imaging sequence: TR = 2.08 ms, TE = 30 ms, FoV = 192 mm, FA = 90°, matrix size 64 × 64, slice thickness = 3 mm, 39 transversal slices.

The behavioral data were evaluated using paired sample *t*-test. The rs-fMRI data were analysed using SPM12 running under Matlab R2015b and preprocessed using realign and unwarp, slice timing correction, and spatial normalization with resampling to 3 × 3 × 3 mm voxels and spatial smoothing (FWHM 6 mm). We controlled data for spatial abnormalities using the tool mask explorer [26] as well as for excessive movement using framewise displacement (FD) with criterion FD < 0.5 mm in less than 10% of scans (nothing excluded) and FD < 1.5 mm in any scans (two sessions excluded). Data was filtered for effects of motion (24 motion parameters).

**Figure 1:**
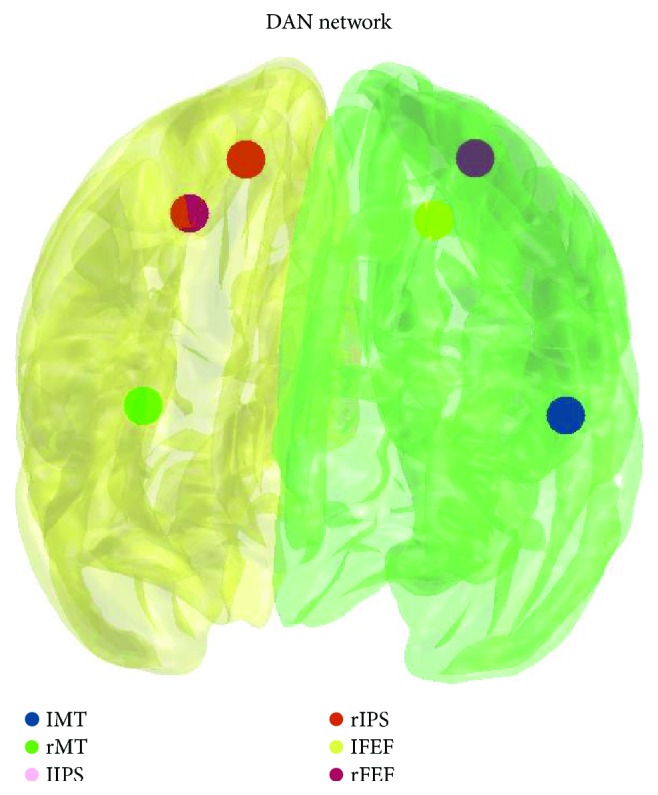
Dorsal attention network: seeds used for the rs-fMRI data analysis. Legend: l/rMT = left/right middle temporal area; l/rIPS = left/right intraparietal sulcus; l/rFEF = left/right frontal eye field.

Seed-based analysis of resting-state data with a seed located at the stimulation site coordinate (a sphere with *r* = 6 mm) was performed using mean as representative signals of the seed [24]. In addition, resting-state functional connectivity of the dorsal attention network (DAN) was analysed. Seeds (a sphere with *r* = 6 mm) were created using 6 coordinates of interest as described in [3], see Figure 1 and Table 1, for the localization of the DAN seeds. Representative mean seed signals were extracted, and correlation matrix was calculated for each subject. Pearson's correlation coefficients were converted using Fisher *r*-to-*z* transformation to *z* values. The average connectivity within DAN was calculated as the mean of *z* values for each seed pair. Wilcoxon signed-rank test was used to assess the change in DAN connectivity induced by the lSPL iTBS stimulation.

## 3. Results

In this exploratory study, TBS was generally well tolerated. There were no adverse effects apart from a mild headache reported by one person. Behavioral results of the Stroop task indicate that the RT after the incongruent stimuli were generally longer than the RT after the congruent stimuli resulting from the longer processing of the conflicting stimuli. The overall task accuracy reached 97%, and it was not influenced by any stimulation protocol (data not shown). We observed a trend toward decreased RT due to iTBS of the lSPL (see Table 2).

In the next step, we further explored neural underpinnings of the abovementioned behavioral changes (some enhancement of cognitive speed induced by iTBS applied over the lSPL) using rs-fMRI analyses. Seed-based analysis of resting-state data showed a significant increase in connectivity after iTBS of lSPL between lSPL and the left cerebellar nodule (−6 −64 −32, *p* = 0.008) and a nearly significant increase between lSPL and the right anterior insula (33 20 −2, *p* = 0.065). Moreover, we observed a significant increase in connectivity within the DAN network (mean value before versus after stimulation: 0.4685 versus 0.5277, *p* = 0.0251).

## 4. Discussion

The current work shows for the first time that even a very short single train of rTMS (intermittent TBS (iTBS)) applied over the left posterior parietal cortex (i.e., an area shown to be activated during the Stroop task performance [5]) may enhance cognitive speed in HYS.

Studies using TBS applied over the areas other than DLPFC and involved in cognitive processing such as IFG or SPL are still missing in the literature. Our team showed that rTMS over the right IFG increased the cognitive processing speed (improvement in all subtests of the Stroop task) in nondemented patients with Parkinson's disease [14] or shortened the cognitive event-related potential (ERP of P3) latency in Parkinson's disease patients with implanted DBS electrodes [23]. rTMS applied over the right IFG in patients with mild cognitive impairment and mild Alzheimer's disease showed significant cognitive improvement in attention and psychomotor speed (Stroop test part Words). Differences in the values before and after stimulation were 4.8 times higher with the IFG stimulation than with the vertex stimulation [7]. Studies using TMS protocols applied over the SPL for enhancement of cognitive functions are sparse. Right parietal rTMS improved visual attention as measured by attentional blink paradigm [27]. Luber et al. [28] showed that 5 Hz rTMS applied during the retention period to the midline parietal cortex speeded reaction times in working memory task without decreasing accuracy. Theta (i.e., 5 Hz) and beta (20 Hz) frequency rTMS to the right parietal cortex enhanced global versus local visual processing, respectively [29].

The lSPL has been the major node of the DAN which is known to be engaged in externally directed conditions and to control processing of visual information while performing a visual cognitive task [3]. Combining rTMS with fMRI showed that iTBS applied over this node enhanced rs-connectivity of the DAN as well as increased connectivity between the stimulated site and other distant brain areas, namely the left cerebellar nodule and the right anterior insula. The cerebellar nodule was shown to be activated by continuous observation of visual stimuli and to have a connection with the frontal eye field, that is, the frontal DAN node [30]. On the other hand, the anterior insula is involved in the frontoparietal control network [3, 31] and heavily interconnected with other prefrontal regions such as the dorsal anterior cingulate cortex, anterior prefrontal regions, and the dorsolateral prefrontal cortex. Activations of the insula together with the abovementioned prefrontal regions are commonly observed with a variety of cognitive control processes such as conflict monitoring, information integration, and response selection [3, 5, 32–35], all of which are involved in the Stroop task. Taken together, our interim data shows that iTBS has an effect particularly on the DAN resting-state functional connectivity and may enhance the cognitive speed of the task performance even in HYS.

We are fully aware of some limitations of this study. We focused solely on the effects of active stimulation applied over specific cortical sites known to be engaged in the task performance. In this exploratory study, we further analysed resting-state functional connectivity changes induced by the TBS protocol and stimulation site with a measurable behavioral aftereffect; that is, we tried to identify neural underpinnings of the iTBS applied over the left SPL. However, the behavioral aftereffect of this specific stimulation was only marginal due to the ceiling effect in cognitively intact healthy young participants, and this is a clear study limitation. Therefore, our results should be interpreted with caution. Despite the fact that the order of the stimulation protocols and stimulation sites was randomised across subjects and sessions and the individual stimulation sessions were separated by at least a one-day interval without stimulation, we cannot exclude the order and carry-over effects completely. This was the exploratory study, and further research controlling for placebo effects is warranted. On the other hand, we for the first time provide evidence for the single-session ultrashort iTBS to induce significant modulatory aftereffects on the rs-fMRI connectivity measures.

## 5. Conclusions

This exploratory study showed that single-session iTBS applied over the lSPL tended to enhance the speed of the Stroop task performance in the HYS group via increased connectivity of the DAN, brain areas that are known to be engaged in the task performance. To assess possible iTBS “treatment” effects, it will be necessary to include multiple-session TBS and focus on healthy seniors and patients with early AD/PD. However, the current results obtained in HYS are promising, and a future set of data in various patient groups may provide a deeper understanding of brain plasticity mechanisms and outline new possibilities for improving cognitive functions in patients with distinct neurodegenerative brain diseases.

## Figures and Tables

**Table 1:** Coordinates of seeds of DAN network, according to Gao and Lin [3].

*X*	*Y*	*Z*	Area	Network
−45	−69	−2	IMT	DAN
50	−69	−3	rMT	
−27	−52	57	lIPS	
24	−56	55	rIPS	
−25	−8	50	lFEF	
27	−8	50	rFEF	

Note: l/rMT = left/right middle temporal area; l/rIPS = left/right intraparietal sulcus; l/rFEF = left/right frontal eye field.

**Table 2:** Summary of the Stroop task response times (expressed as mean ± SD and tested using paired-sample *t*-test).

Area	Protocol	Target	Before	After	Difference	*p* value
rIFG	cTBS	CON	410.6 ± 80.6	406.1 ± 71.1	−4.6 ± 21.3	0.351
		INCON	415.2 ± 75.4	414.0 ± 71.2	−1.2 ± 16.9	0.750
		*p* value	0.345	0.014	0.422	

rIFG	iTBS	CON	426.3 ± 103.5	426.3 ± 109.6	0.0 ± 32.2	0.999
		INCON	429.7 ± 110.7	420.4 ± 112.2	−9.3 ± 32.5	0.218
		*p* value	0.326	0.123	0.057	

lSPL	cTBS	CON	418.6 ± 90.2	406.6 ± 79.8	−12.0 ± 30.7	0.096
		INCON	423.0 ± 92.2	412.4 ± 78.2	−10.6 ± 27.2	0.098
		*p* value	0.200	0.138	0.775	

lSPL	iTBS	CON	412.0 ± 81.7	397.8 ± 64.7	−14.1 ± 31.7	**0.060**
		INCON	418.8 ± 83.8	404.3 ± 68.1	−14.6 ± 32.0	**0.056**
		*p* value	0.030	0.039	0.888	

Note: rIFG = right inferior frontal gyrus; lSPL = left superior parietal lobule; cTBS = continuous theta burst stimulation; iTBS = intermittent theta burst stimulation; CON = congruent stimuli; INCON = incongruent stimuli. Trends towards enhanced cognitive speed enhanced by the stimulation (although nonsignificant) are depicted in bold.
